# Enthusiasm-Based or Evidence-Based Charities: Personal Reflections Based on the Project P.A.T.H.S. in Hong Kong

**DOI:** 10.1100/tsw.2008.111

**Published:** 2008-08-27

**Authors:** Daniel T. L. Shek

**Affiliations:** ^1^ Centre for Quality of Life, Hong Kong Institute of Asia-Pacific Studies, The Chinese University of Hong Kong, Hong Kong; ^2^ Kiang Wu Nursing College of Macau, Macau; ^3^ Social Welfare Practice and Research Centre, The Chinese University of Hong Kong, Hong Kong

**Keywords:** evidence-based practice, scientific evidence, charitable foundations, funding allocation, Project P.A.T.H.S.

## Abstract

In charitable foundations throughout the world, different approaches are used to allocate funding. As many projects with good will (i.e., enthusiasm-based charity) actually fail to help those who really need it, it is argued that the evidence-based approach (i.e., charity guided by scientific evidence) represents the best strategy to support projects that can really help the needy. Using this approach, scientific research findings are systematically used to (1) understand the nature of the problem and/or social needs, (2) design appropriate intervention programs based on the best available evidence, and (3) systematically evaluate the outcomes of the developed program. Using the Project P.A.T.H.S. funded by the Hong Kong Jockey Club Charities Trust as an example, the characteristics underlying this approach are outlined. The systematic use of scientific evidence in the Project P.A.T.H.S. is exemplary in different Chinese societies. This project provides much insight for charitable foundations and funding bodies locally and globally.

## INTRODUCTION

In different parts of the world, charitable foundations have been set up by businessmen, corporations, and the public in order to help people in need. Most of the time, such foundations are seen as a sign of corporate social responsibility and an act of the company to serve society. Some charitable organizations outside Hong Kong include the Bill and Melinda Gates Foundation[1], Ford Foundation[2], and Rockefeller Foundation[3]. Similarly, there are many charitable foundations established in the private sector to meet the social needs of Hong Kong. Some examples include the Li Ka Sing Foundation, Hong Kong Bank Foundation, and Sun Hung Kai Properties-Kwok's Foundation. There are also foundations established by the Government to support welfare, education, and community projects, such as the Beat Drugs Fund and the Quality Education Fund.

Another important charitable organization in Hong Kong is The Hong Kong Jockey Club Charities Trust of The Hong Kong Jockey Club[4]. As one of the largest racing organizations in the world, The

Hong Kong Jockey Club is the sole authorized operator of horse race and football betting in Hong Kong. On behalf of the Hong Kong Lotteries Board, it also manages the Mark Six lotteries. One unique feature of The Hong Kong Jockey Club is that it upholds the not-for-profit gambling policy with the donation of surplus to charity and community projects[5]. In the past decade, The Trust donated an average of more than HK$1 billion each year. In 2006/07, a total of HK$1.05 billion was donated by the Trust to support 107 charities and community projects. These include community services projects (e.g., "CADENZA", a project serving old people, and Project "P.A.T.H.S. to Adulthood", a project serving young people); education and training projects (e.g., The Hong Kong Jockey Club Education Fund and "READ & Write: A Jockey Club Learning Support Network", a project serving people with specific learning difficulties); medical and health projects (e.g., establishment of the Centre for Health Protection); and sports, recreational, and cultural projects (e.g., establishment of the Jockey Club Kau Sai Chau Public Golf Course and a joint Sports Medicine and Health Sciences Centre with the involvement of The Chinese University of Hong Kong and The Hong Kong Polytechnic University).

## FUND ALLOCATION POLICIES IN CHARITABLE FOUNDATIONS

It is both academically and practically important to know how funds are allocated in different charitable foundations and funding bodies. Based on the author's involvement in different high-level advisory committees and grant allocation committees of the Government of the Hong Kong Special Administrative Region, P.R.C., and experiences in applying funding for welfare agencies from different charitable foundations, there are roughly seven approaches (some of which are not mutually exclusive) adopted by different charitable foundations and funding bodies in vetting applications and allocating funding:

- *Ease-based allocation approach*: Under this approach, funds are allocated based on convenience. Generally speaking, the grants available for allocation in a particular year will be allocated to as many applications as possible. One advantage of this approach is that it can guarantee that all applicants are happy because everybody gets something and the available money for allocation will be used up. One obvious disadvantage of this approach is that some applications with low quality may still get funding.
- *Eccentric and erratic allocation approach*: Under this approach, funds allocated are not based on consistent and rational principles. Most of the time, funding allocation depends on the taste and preference of the board members as well as the chairman of the board. As a result, funding allocation is often a product of politics and dynamics within the board rather than rational decision making.
- *Echo-based or envy-based allocation approach*: Utilizing this approach, funding allocation is based on a "follow-suit" manner. For example, if poverty is the main focus of society recently and other charitable foundations fund projects on helping poor people, the foundation will fund similar projects. In addition, as there is overt and/or covert competition among different charitable foundations, a foundation may envy what other foundations are doing, hence funding projects of similar nature for an unhealthy competition purpose.
- *Exposure-based allocation approach*: As some business corporations may regard publicity as the sole purpose of setting up charitable foundations, they may simply fund those projects that can bring public attention and promote exposure of the company. Under this approach, whether a project can serve as a good vehicle for publicity is the prime consideration in funding allocation.
- *Enthusiasm-based or emotion-based allocation approach*: For some social problems, such as poverty or mental retardation, the miserable conditions of the clients will trigger the passion and emotions of the board members of charitable foundations. As such, the vetting committee may fund programs that claim to be able to improve the miserable conditions of the needy. In fact, programs may simply be funded out of enthusiasm and passion. Of course, developing programs to meet the needs of those living in miserable conditions is important. However, overattention paid to the miserable conditions of the needy people at the expense of the real ability of the proposed program to improve the conditions of the needy may not be helpful to the needy at the end of the day. Obviously, we may have passion and good will, but the proposed program may be a futile program. As pointed out by the Bill and Melinda Gates Foundation, “philanthropy plays an important but limited role”, but “science and technology have great potential to improve lives around the world”[1].

- *Error-based allocation approach*: Utilizing this approach, the funding body funds programs that have been proved to be ineffective or programs that are not supported by evaluation findings. To avoid using this approach, adequate understanding of the literature on intervention research in human services is indispensable. Some examples will be given below to illustrate this approach.
- *Evidence-based allocation approach*: To help the needy in an effective manner, we must understand the nature of their problem and need, develop an intervention program based on best-available evidence, and systematically evaluate the effectiveness of the implemented program. Obviously, science and scientific evidence play an important role in guiding charitable foundations to make important decisions. This approach is used in some of the major foundations. For example, the Rockefeller Foundation explicitly states that “the Foundation vigorously and regularly measures impact and outcomes. Our initiatives specify clear time frames, identify anticipated results, and require monitoring and evaluation. This does not mean that we expect to solve the world’s thorniest problems overnight. It does mean that Foundation-supported work defines hypotheses, articulates both short- and longer-term objectives, foresees and adapts to changing circumstances, and fully integrates verifiable methods of assessing progress”[3].

## ENTHUSIASM-BASED AND ERROR-BASED ALLOCATION VS. EVIDENCE-BASED FUNDING POLICIES

In the literature on program evaluation, there are numerous examples showing that programs based on good will and passion may not be effective programs. Two examples are given here to illustrate the problems of enthusiasm-based and error-based allocation approaches. The first example is the Project D.A.R.E. (Drug Abuse Resistance Education) Program. This program was founded in 1983 in Los Angeles and was implemented in 75% of the school districts in the U.S. and in more than 43 countries around the world to help young people to resist pressure, and to live drug- and violence-free lives. Unfortunately, several longitudinal studies have repeatedly shown that the program did not work[6,7]. As pointed out by Rosenbaum and Hanson[8], “…can this popular school-based program prevent drug use at the stages in adolescent development when drugs become available and are widely used, namely, during the high school years? Unfortunately, the answer to the question is ‘no’” (p. 404). Obviously, popularity and enthusiasm alone do not guarantee program success.

The second example is programs that involve visits to prisons so as to deter young people from committing crimes. In such programs (e.g., Scared Straight), it is typically assumed that the organization of visits to prison facilities for juvenile delinquents and children at risk of delinquency would deter the likelihood of offence, hence lowering the crime rates for the young people concerned[9]. Unfortunately, the review conducted by Petrosino et al.[10] showed that such programs could not deter young people from committing crimes. In fact, the reviewers concluded that “programmes like ‘Scared Straight’ are likely to have a harmful effect and increase delinquency relative to doing nothing at all to the same youths. Given these results, agencies that permit such programmes must rigorously evaluate them not only to ensure that they are doing what they purport to do (prevent crime) – but at the very least they do not cause more harm than good” (p.2).

Rubin and Babbie[11] pointed out that there are many ways of generating knowledge claims. First, we develop knowledge claims via tenacity, such as cultural beliefs. One example is the cultural belief that “a dutiful son is a product of the rod”, which endorses the importance of harsh punishment in child rearing. The second way is common sense, such as venting emotion is important for an emotionally distressed person. The third way is to derive knowledge claims based on authority, such as university professors and Government officials. For example, under the influence of Newton, people believe that there are physical laws that are universal in any context (but these universal laws become shaky under Einstein's theory). The fourth way is to develop knowledge claims through a priori method or logic. Based on a certain premise (e.g., poverty leads to juvenile delinquency), one can logically deduce certain knowledge claims (e.g., juvenile delinquency is higher in poor relative to nonpoor adolescents). Finally, knowledge claims can be derived from the scientific method. Through induction and deduction, one can verify the validity of the deduced hypotheses. Among these methods of developing knowledge claims, the scientific method is the preferred approach of generating knowledge claims.

According to Gambrill[12], there are two ways of understanding the world and generating knowledge claims. The first way is authority-based practice that generates authority-based knowledge. Under this approach, there is reliance on authority (such as experts and authority in the field) as the ultimate standard and false knowledge where belief that is not true and not questioned is generated. In addition, authority-based practice is, in fact, pseudoscience with several characteristics, including discouraging critical scrutiny of knowledge claims, using rituals of science without substance, relying on anecdotal experience, lacking skepticism, treating open minds as uncritical minds, ignoring negative evidence, and upholding beliefs that are not verifiable.

On the other hand, evidence-based practice emphasizes the importance of utilizing evidence in providing service that usually involves five steps[13]. In Step 1, issues related to intervention are formulated in terms of answerable questions. In Step 2, the practitioner searches the literature to locate the best evidence on the problem under focus. In Step 3, the worker critically appraises the evidence in terms of its validity, reliability, and clinical applicability. In Step 4, the best evidence that has been critically scrutinized is implemented. In Step 5, clinical performance is evaluated. Gambrill[14] further points out that there are several core values underlying the intellectual base of evidence-based practice. These include courage (evaluate knowledge claims in a bold manner), curiosity (deep interest in knowledge), intellectual empathy (accurately presenting the views of others), humility (acknowledging one's limitations), integrity (no double standards), and persistence (endurance in view of adversity). Gambrill[12] clearly states that "different ways of knowing differ in the extent to which they highlight uncertainty and are designed to weed out biases and distortions that may influence assumptions" (p. 343). Obviously, systematic use of research findings in understanding the problem encountered (e.g., adolescent substance abuse), developing appropriate intervention programs, and collecting evaluation data in a systematic manner to assess the implementation of the program and outcomes are important.

## PROJECT P.A.T.H.S. AND EVIDENCE-BASED POSITIVE YOUTH DEVELOPMENT PROGRAM

A survey of the literature shows that there are worrying trends and phenomena related to the development of adolescents in Hong Kong, such as mental health problems, abuse of psychotropic substances, adolescent suicide, school violence, and drop in family solidarity[15]. As such, primary prevention programs that target specific adolescent developmental problems and positive youth development programs are called for. However, research findings show that there are very few systematic and multiyear positive youth development programs in Hong Kong. Even if such programs exist, they commonly deal with isolated problems and issues in adolescent development (i.e., deficits-oriented programs), and they are relatively short term in nature. In addition, systematic and long-term evaluation of the available programs does not exist.

To promote holistic development among adolescents in Hong Kong, The Hong Kong Jockey Club Charities Trust approved HK$400 million to launch a project entitled "P.A.T.H.S. to Adulthood: A Jockey Club Youth Enhancement Scheme". The word "P.A.T.H.S." denotes **P**ositive **A**dolescent **T**raining through **H**olistic **S**ocial Programmes. The Trust invited academics of five universities in Hong Kong to form a Research Team with The Chinese University of Hong Kong as the lead institution and the author as the Principal Investigator in order to develop a multiyear universal positive youth development program to promote holistic adolescent development in Hong Kong.

To achieve these objectives, a curricula-based program (Tier 1 Program) was developed. The design of the program and constructs included in the program were based on a thorough and systematic review of the scientific literature[16]. In particular, focus was put on the work of Catalano et al.[17], who found that only 25 out of 75 positive youth development programs under review were successful, and 15 positive youth development constructs were identified in the successful programs. These constructs are presented as follows:

1. Promotion of Bonding: Developing the program participants' relationship with healthy adults and positive peers in the extrafamilial context (school, community, and cultural contexts), and caregivers and significant others in the intrafamilial context.
2. Cultivation of Resilience: Promoting the capacity for adjusting to change and stressful events in healthy and adaptive ways.
3. Promotion of Social Competence: Training the program participants' interpersonal skills (including communication, assertiveness, refusal and resistance, conflict resolution and interpersonal negotiation) and providing opportunities to practice such skills.
4. Promotion of Emotional Competence: Training the program participants' skills to recognize feelings in oneself and others (including empathy), skills to express feelings, skills to manage emotional reactions or impulses (delay of gratification and frustration tolerance), and emotional self-management strategies.
5. Promotion of Cognitive Competence: Developing the program participants' cognitive abilities, processes, or outcomes, including academic performance, logical thinking, critical thinking, problem solving, decision making, planning and goal setting, and self-talk.
6. Promotion of Behavioral Competence: Cultivating verbal communication (making requests and positive response to criticisms) and nonverbal communication, and taking action skills and providing reinforcement for the effective behavior choices and action pattern.
7. Promotion of Moral Competence: Developing a sense of right and wrong, and respect for rules and standards as well as social justice.
8. Cultivation of Self-Determination: Promoting the program participants' sense of autonomy, independent thinking, or self-advocacy.
9. Promotion of Spirituality: Helping the program participants to develop purpose and meaning in life, hope, or beliefs in a higher power.
10. Development of Self-Efficacy: Promoting the program participants' coping and mastery skills and changing their negative self-efficacy expectancies or self-defeating cognitions.
11. Development of Clear and Positive Identity: Promoting healthy identity formation and achievement, including positive identification with one's social or ethnic identity.
12. Promotion of Beliefs in the Future: Helping program participants to develop future potential goals, choices, or options.
13. Provision of Recognition for Positive Behavior: Developing systems for rewarding, recognizing, or reinforcing participants' positive behavior, such as prosocial behavior or positive changes in behavior.
14. Providing Opportunities for Prosocial Involvement: Designing activities and events for program participants to make positive contribution to groups.
15. Fostering Prosocial Norms: Encouraging program participants to develop clear and explicit standards for prosocial engagement.

Furthermore, based on the literature review, several assertions are maintained in the conceptual model underlying the program in the present project[16].

As far as curriculum development is concerned, the following principles are maintained in the process of designing the Tier 1 Program after a thorough review of the literature[18]:

- Principle 1: The program is a comprehensive universal program that utilizes a wide range of positive youth development constructs that have been identified in the effective programs.
- Principle 2: Relevant theoretical models and research findings in both Western and Chinese contexts are used to guide the development of the program.
- Principle 3: Holistic adolescent development in different domains (physical, psychological, social, and spiritual domains) is focused upon.
- Principle 4: Both adolescent developmental assets and developmental problems (e.g., drug, sex, delinquency, violence, lifestyle, money spending, and mental health issues) are considered in the process.
- Principle 5: The proposed program content is developmentally appropriate.
- Principle 6: Relevant cultural elements are included in the program.
- Principle 7: Multiyear intervention programs rather than one-shot programs are designed.
- Principle 8: Proper and adequate training is planned for the teachers and social workers who implement the programs.
- Principle 9: Relevant teaching strategies and methods (e.g., using peers to demonstrate skills and change norms) are used to maximize the learning effects.
- Principle 10: Active participation and involvement of the students are emphasized.
- Principle 11: Besides classroom activities, programs outside the classroom are developed.
- Principle 12: Generalization of the competence developed to the real-life world is emphasized.
- Principle 13: Students are involved in the design of the program activities.
- Principle 14: Relevant issues (e.g., gender differences, school differences, and class differences) are considered in the program design.
- Principle 15: Besides changing the students, attempts to change the families (e.g., encouraging parental involvement) and schools (e.g., school improvement and reorganization initiatives included) are included.
- Principle 16: Ongoing evaluation at all stages is carried out.

As training of potential program implementers is an important part of the project, training programs were also designed based on a thorough review of the literature. Based on a critical review of the studies in this area, several principles governing training of the potential program implementers are maintained in the study[19]:

- Principle 1: Design of training program is based on training theories/models.
- Principle 2: Acquisition of knowledge about adolescents and the program in the training program.
- Principle 3: Acquisition of knowledge about the curriculum structure of the program in the training program.
- Principle 4: Cultivation of appropriate implementation skills in the training program.
- Principle 5: Cultivation of self-reflection skills in the training program.
- Principle 6: Encouragement of workers to be role models.
- Principle 7: Promotion of motivation of the trainees.
- Principle 8: Promotion of self-efficacy of the trainees.
- Principle 9: Provision of opportunities for demonstration and practice.
- Principle 10: Provision of adequate training time.
- Principle 11: Consideration of cultural context in the design of training program.
- Principle 12: Evaluation of training program.

Finally, based on literature review, an evaluation design based on multiple evaluation strategies and the principle of triangulation was adopted with the following evaluation methods[20]:

1. Objective Outcome Evaluation: Evaluation findings based on a one-group pre-/post-test design[21] and a randomized group trial were collected.
2. Subjective Outcome Evaluation: Both students and program implementers were invited to complete subjective outcome evaluation forms after completion of the program[22,23]. Convergence between subjective outcome evaluation and objective outcome evaluation findings was also examined[24].
3. Process Evaluation: Systematic observations were carried out in randomly selected schools to understand the program implementation details[25].
4. Interim Evaluation: To understand the process of implementation, interim evaluation was conducted by randomly selecting half of the participating schools[26].
5. Qualitative Evaluation (Focus Groups Based on Students): Focus groups involving students based on schools randomly selected from the participating schools were carried out[27].
6. Qualitative Evaluation (Focus Groups Based on Program Implementers): Focus groups involving instructors based on schools randomly selected from the participating schools were carried out.
7. Qualitative Evaluation (In-depth Interviews with Program Implementers): Prolonged in-depth interviews with two teachers were carried out.
8. Qualitative Evaluation (Case Study Based on Focus Groups): A case study based on seven schools participating in the Secondary 1 Program of the Full Implementation Phase was conducted.
9. Qualitative Evaluation (Student Logs): Four students were invited to reflect on their experiences after joining the classes and application of things learned to real life.
10. Qualitative Evaluation (Student Products): Students' weekly diaries were collected after completion of the program[28].

An integration of research findings collected from different sources shows that there is support for the effectiveness of the P.A.T.H.S. Project. The details of the evaluation finding supporting the Project P.A.T.H.S. can be seen in the website of the project (http://www.paths.hk).

## TOWARDS EVIDENCE-BASED WELFARE AND HUMAN SERVICE IN HONG KONG

Shek et al.[29] showed that evidence-based social work practice was very primitive in Hong Kong and there were many obstacles involved. Of course, one good way to promote evidence-based practice is for charitable foundations to adopt an evidence-based approach in vetting and funding research proposals and attempts that systematically utilize research findings in understanding the problem area, developing evidence-based programs, and evaluating the effectiveness of the developed programs. As members of the funding allocation committees in charitable foundations and other funding bodies, one should realize that there are six possible types of community and welfare programs with different levels of effectiveness: (1) ineffective or harmful intervention, (2) intervention unlikely to be beneficial, (3) intervention with unknown effectiveness, (4) intervention with both benefits and adverse effects, (5) intervention likely to be beneficial, and (6) intervention that is effective, which is reflected by clear evidence from controlled trials. For funding bodies and charitable foundations, it is important to fund projects routinely that are promising and systematically utilize research findings to understand the nature of the issue to be tackled, develop the intervention program, and evaluate the program. Obviously, the Project P.A.T.H.S. can be regarded as an exemplary program that systematically can instill paradigm shifts in charitable foundations and funding bodies locally and globally.

## ACKNOWLEDGMENTS

The preparation for this paper and the Project P.A.T.H.S. were financially supported by the Hong Kong Jockey Club Charities Trust.
